# Effects of transcutaneous vagus nerve stimulation in individuals aged 55 years or above: potential benefits of daily stimulation

**DOI:** 10.18632/aging.102074

**Published:** 2019-07-30

**Authors:** Beatrice Bretherton, Lucy Atkinson, Aaron Murray, Jennifer Clancy, Susan Deuchars, Jim Deuchars

**Affiliations:** 1 School of Biomedical Sciences, Faculty of Biological Sciences, University of Leeds, Leeds LS2 9JT, UK; 2 School of Life Sciences, University of Glasgow, Glasgow G12 8QQ, UK

**Keywords:** vagus nerve stimulation, autonomic nervous system, neuromodulation, quality of life, mood

## Abstract

Ageing is associated with attenuated autonomic function. Transcutaneous vagal nerve stimulation (tVNS) improved autonomic function in healthy young participants. We therefore investigated the effects of a single session of tVNS (studies 1 and 2) and tVNS administered daily for two weeks (study 3) in volunteers aged ≥ 55 years. tVNS was performed using modified surface electrodes on the tragus and connected to a transcutaneous electrical nerve stimulation (TENS) machine. Study 1: participants (n=14) received a single session of tVNS and sham. Study 2: all participants (n=51) underwent a single session of tVNS. Study 3: participants (n=29) received daily tVNS for two weeks. Heart rate variability and baroreflex sensitivity were derived. Quality of life (QoL), mood and sleep were assessed in study 3. tVNS promoted increases in measures of vagal tone and was associated with greater increases in baroreflex sensitivity than sham. Two weeks of daily tVNS improved measures of autonomic function, and some aspects of QoL, mood and sleep. Importantly, findings showed that improvements in measures of autonomic balance were more pronounced in participants with greater baseline sympathetic prevalence. This suggests it may be possible to identify individuals who are likely to encounter significant benefits from tVNS.

## Introduction

Ageing is associated with changes in autonomic nervous system function and is characterized by increases in sympathetic and decreases in parasympathetic nervous activity [1,2]. Such autonomic changes can be detrimental to heart function, emotion, mood and gut function, and may play a role in a range of conditions that increase in prevalence with ageing, including heart failure [3,4], hypertension [5] and depression [6,7]. These conditions are typically accompanied with increases in medication consumption and decreases in quality of life (QoL). Preventing or reducing age-related changes in autonomic balance may therefore improve health in older individuals, as well as increase their independence, QoL and mood (particularly depression). Potential benefits include reduced risk of mortality and reductions in the need for medication and/or hospitalisation.

Interventions which aim to boost parasympathetic activity and/or decrease sympathetic activity include vagus nerve stimulation (VNS) and transcutaneous vagal nerve stimulation (tVNS). VNS involves surgically implanting an electrode around the cervical vagus nerve and a generator unit in the thoracic wall [8]. However, due to its invasive nature, technical complications and side-effects e.g. pain, coughing, hoarseness of voice [8,9], potentially simpler and safer therapies are of interest.

tVNS is a simple, non-invasive and inexpensive therapy that involves stimulating the auricular branch of the vagus nerve (ABVN) at outer parts of the ear, conferring autonomic benefits in healthy volunteers [10]. For instance [11], revealed that 15 minutes of tVNS administered to the tragus significantly increased heart rate variability (HRV), at least partly through reductions in sympathetic nerve activity. An increase in parasympathetic activity is also likely, since tVNS increases spontaneous cardiac baroreflex sensitivity (BRS) even in healthy young males [12]. Baseline HRV decreases with increasing age [13–16] and since tVNS induces larger increases in HRV in participants with lower starting HRV [11], tVNS could be particularly effective in older compared to younger participants. We therefore investigated if tVNS could ameliorate age-related changes in autonomic function.

We report on three studies into the effects of tVNS in participants aged ≥ 55 years. In the first study we compared the effects of acute tVNS on cardiovascular autonomic function with the effects of sham stimulation by measuring HRV and BRS. Since not all participants responded to tVNS, we examined if it was possible to identify potential tVNS responders from baseline parameters. In the second study, we explored the effects of acute tVNS on autonomic function in the same age group by expanding the sample. The final study aimed to examine how daily tVNS (a 15-minute session administered once daily for two weeks) impacted measures of autonomic function, as well as health-related QoL, mood and sleep.

## RESULTS

### Study 1

14 participants aged ≥ 55 years with no previous medical history of hypertension, cardiac disease, diabetes mellitus or epilepsy were enrolled on the study (see Supplementary Table 1).

### tVNS *vs.* sham effects on cardiac baroreflex and HRV

**Figure 1:**
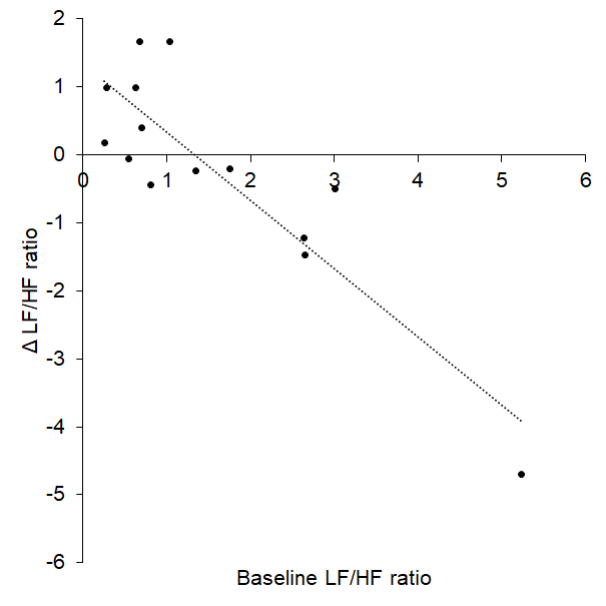
Baseline LF/HF ratio significantly predicted response (change) to tVNS.

Change in baroreflex sensitivity (BRS) between baseline and tVNS significantly differed between the tVNS and sham visits (p = 0.028): there was a significantly greater increase in BRS during the tVNS visit (3.28 ± 0.59 ms/mmHg) compared to the sham visit (0.81 ± 0.68 ms/mmHg). Baseline heart rate variability (HRV), measured as ratio of LF/HF power, significantly predicted response to tVNS (R^2^ = 0.772, p < 0.001, see Figure 1), where higher resting LF/HF ratio was associated with greater decreases during tVNS. Removing the potential outlier with a baseline LF/HF ratio > 5 had a slight impact on the regression (R^2^ = 0.480, p = 0.009). This HRV analysis revealed that the LF/HF ratio response of four participants was greater than a 20% increase, corresponding to a definition of responders previously applied [11].

### Study 2

Fifty-one healthy participants aged ≥ 55 years were recruited. Two participants were excluded due to the presence of > 3 ectopic heartbeats in any given five-minute period (n = 1 female). A further participant was excluded due to a respiration rate < 10 breaths per minute (n = 1 male). Characteristics of the final sample for study 2 are presented in Supplementary Table 2.

### tVNS significantly increased heart rate variability in ≥ 55-year-old participants

Analysis of the whole cohort in study 2 revealed that measures considered to reflect vagal activity were significantly higher during tVNS (RMSSD: p = 0.007; pRR50: p = 0.005; SD1: p = 0.007; BRS: p = 0.001) and recovery (HF power: p = 0.008) compared to baseline (see Supplementary Table 3).

In addition, LF power, SD2 and nSD2, representing combined sympathetic and parasympathetic influences on the heart, were significantly higher during tVNS (p = 0.007, p < 0.001 and p = 0.005 respectively) and recovery (p = 0.001, p < 0.001 and p = 0.002 respectively).

Measures of overall variability were significantly impacted by tVNS. Total power, mean RR interval, Δ RR, SDRR and S were significantly higher during tVNS (all p < 0.001) and recovery (all p < 0.001) compared to baseline.

### Response to tVNS was predicted by baseline LF/HF ratio

**Figure 2:**
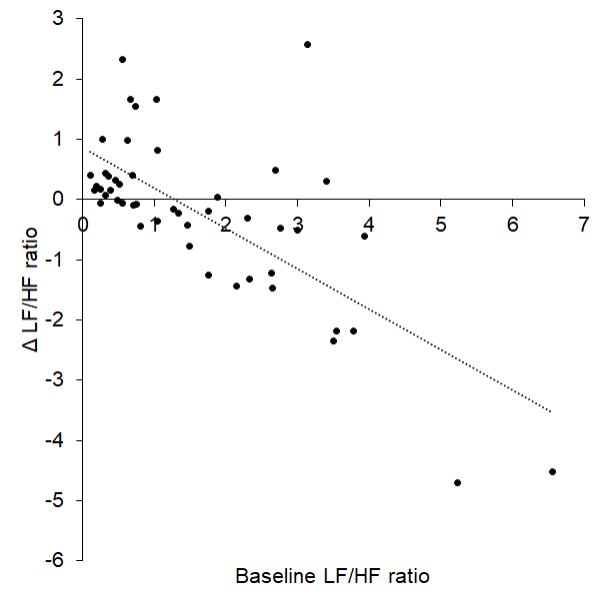
Baseline LF/HF ratio significantly predicted change in LF/HF during tVNS (Δ LF/HF ratio).

Linear regression revealed a significant prediction between baseline LF/HF ratio and Δ LF/HF ratio such that a higher LF/HF ratio predicted a greater decrease to tVNS (R^2^ = 0.498, p < 0.001, Figure 2) in the cohort of 51 participants.

### tVNS responders showed shifts towards parasympathetic prevalence during tVNS

Using the response definition employed in study 1 (20% decrease in LF/HF ratio), 16 responders and 32 non-responders were identified. Further analysis revealed that baseline measures reflecting parasympathetic activity (HF power: p = 0.013; nuHF: p = 0.001; RMSSD: p = 0.029; SD1: p = 0.029; nSD1: p = 0.029 and BRS: p = 0.011) and overall variability in HR (S: p = 0.034) were significantly lower in responders compared to non-responders (see Supplementary Table 4). In addition, measures of baseline sympathovagal outflow (nuLF: p = 0.001) and sympathovagal balance (LF/HF ratio: p < 0.001) were significantly higher in responders compared to non-responders. This suggests that at rest, responders had significantly lower vagal tone and greater sympathetic prevalence compared to non-responders. There were no statistically significant differences in demographic information between responders and non-responders (see Supplementary Table 5).

### Study 3

The characteristics of the final sample of 26 participants are presented in Table 1.

**Table 1:** Summary of characteristics of the final sample of study 3.

Final sample size (n)	26
Gender (frequency of males)	9
Age (yrs.)	64.12 (1.02)
BMI (kg/m^2^)	28.07 (1.28)
Baseline systolic blood pressure (SBP, mmHg)	123.67 (3.14)
Baseline diastolic blood pressure (DBP, mmHg)	81.85 (1.92)
Baseline mean arterial pressure (MAP, mmHg)	95.79 (2.22)

### Daily tVNS sessions improved baseline measures of autonomic function

**Figure 3:**
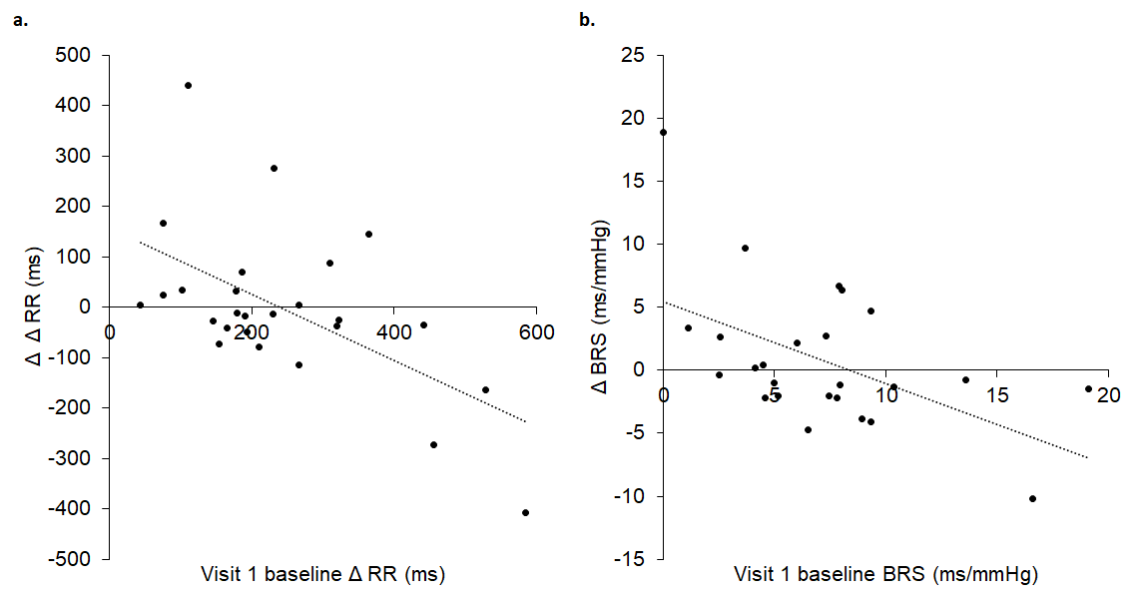
Visit 1 baseline Δ RR (**A**) and BRS (**B**) significantly predicted change at visit 2 baseline, where lower baseline Δ RR (A) and BRS (B) in visit 1 were associated with greater increases in baseline Δ RR (A) and BRS (B) in visit 2. In A, Δ Δ RR reflects the difference between maximum and minimum RR intervals (Δ RR) between visit 1 baseline and visit 2 baseline. In B, Δ BRS reflects the difference in BRS between visit 1 baseline and visit 2 baseline.

Baseline Δ RR, an indicator of cardiac vagal tone, (R^2^ = 0.330, p = 0.002) and BRS (R^2^ = 0.330, p = 0.002) during visit 1 significantly predicted change during baseline at visit 2: the lower the baseline Δ RR and BRS in visit 1, the greater the increase in baseline Δ RR and BRS at visit 2 (Figure 3).

### Measures of autonomic tone after two weeks of daily tVNS

Two-way repeated measure ANOVAs revealed that measures of vagal tone: RMSSD, pRR50, SD1 and nSD1, were significantly higher during visit 2 compared to visit 1 when values during all three recordings (baseline, tVNS, recovery) were combined across each visit (main effect of visit: RMSSD, p = 0.016; pRR50, p = 0.010; SD1, p = 0.016; nSD1, p = 0.022, see Supplementary Table 6). Also, HF power tended to be higher during visit 2 than during visit 1 (main effect of visit: p = 0.051). Similarly, measures reflecting short and long terms variations in HR, total power, SDRR, SD2 and S were significantly greater during visit 2 than visit 1 (main effect of visit: total power, p = 0.046; SDRR, p = 0.024; SD2, p = 0.035 and S, p = 0.012).

Two-way repeated measure ANOVAs also showed that total power (p = 0.010), SDRR (p = 0.001), SD2 (p = 0.001), S (p = 0.017), mean RR interval (p < 0.001) and nSD2 (p = 0.001) were significantly higher during tVNS compared to baseline (main effect of condition). Some of these changes persisted into the recovery period: total power (p = 0.034), SDRR (p = 0.008), SD2 (p = 0.004), mean RR interval (p = 0.005) and nSD2 (p = 0.008) were significantly higher during recovery than in baseline. LF power was also significantly higher during tVNS compared to baseline (p = 0.033, main effect of recording: p = 0.039, see Supplementary Table 7).

**Figure 4:**
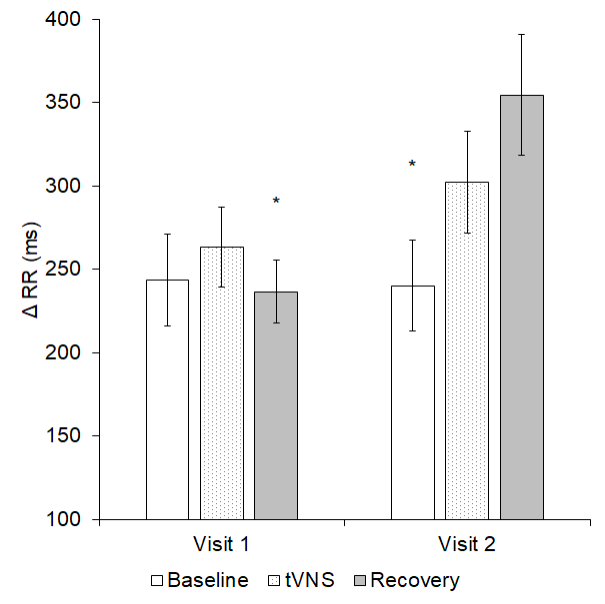
Δ RR significantly differed both within and between the two visits (p = 0.036); * = significantly different to visit 2 recovery.

A significant interaction effect transpired for Δ RR (p = 0.036, see Figure 4). Further analysis revealed that Δ RR was significantly higher during visit 2 recovery compared to visit 1 recovery (p = 0.001). Furthermore, in visit 2 only, Δ RR was significantly greater during recovery than baseline (p = 0.024).

### Response to tVNS in both visits was predicted by baseline LF/HF ratio

**Figure 5:**
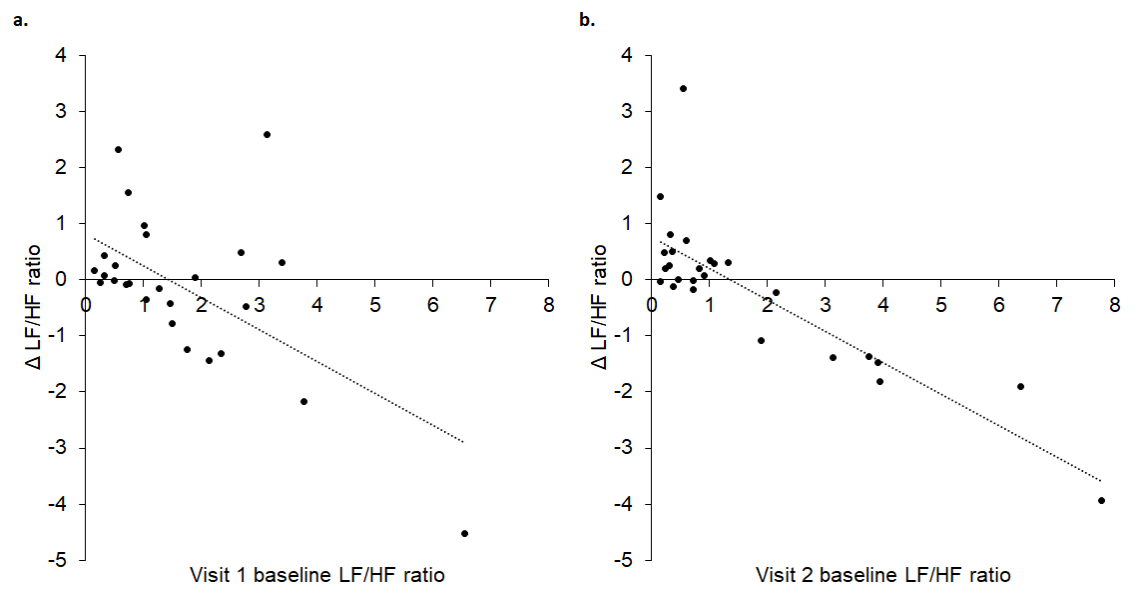
Baseline LF/HF ratio in visits 1 (**A**) and 2 (**B**) significantly predicted change in LF/HF ratio between baseline and tVNS. Δ refers to the differences between baseline and tVNS.

Linear regressions revealed a statistically significant prediction between baseline LF/HF ratio and change during tVNS in visits 1 (R^2^ = 0.352, p = 0.001) and 2 (R^2^ = 0.697, p < 0.001). As illustrated in Figure 5, higher baseline LF/HF ratio values were associated with greater decreases in LF/HF ratio during tVNS in both visits.

### Responders had higher baseline sympathetic prevalence than non-responders

Nine responders and 17 non-responders were identified in visit 1. Responders had significantly lower indicators of vagal tone at baseline in visit 1 than non-responders: HF power (p = 0.034), nSD1 (p = 0.039) and BRS (p = 0.036, see Supplementary Table 8). In addition, responders had significantly higher sympathetic prevalence during visit 1 baseline than non-responders: SD2 (p = 0.028) and nSD2 (p = 0.036), and significantly lower overall variability in HR during visit 1 baseline compared to non-responders: SDRR (p = 0.038) and S (p = 0.039). There were no significant differences in demographic, health-related QoL, mood or sleep characteristics between responders and non-responders (p > 0.05).

**Figure 6:**
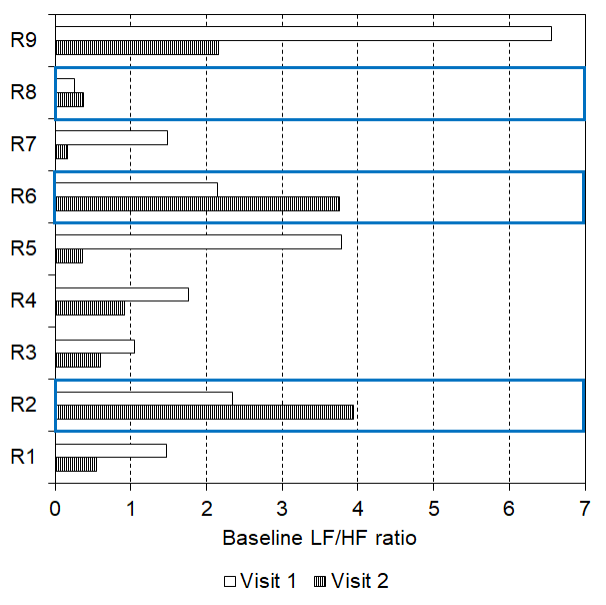
Baseline LF/HF ratio reduced after 2 weeks of daily tVNS in six responders with three showing an increase in baseline LF/HF ratio following the daily tVNS (indicated by the blue boxes).

Interestingly, two-thirds (n = 6) of responders encountered a lowering of their baseline LF/HF ratio after two weeks of daily tVNS (see Figure 6, visit 1 mean: 2.68, SEM: 0.87; visit 2 mean: 0.79, SEM: 0.29, p = 0.028). However, there were three responders whose baseline LF/HF ratio increased between visit 1 (mean: 1.58, SEM: 0.54) and visit 2 (mean: 2.69, SEM: 0.95, see Figure 6), although all still responded in visit 2.

**Figure 7:**
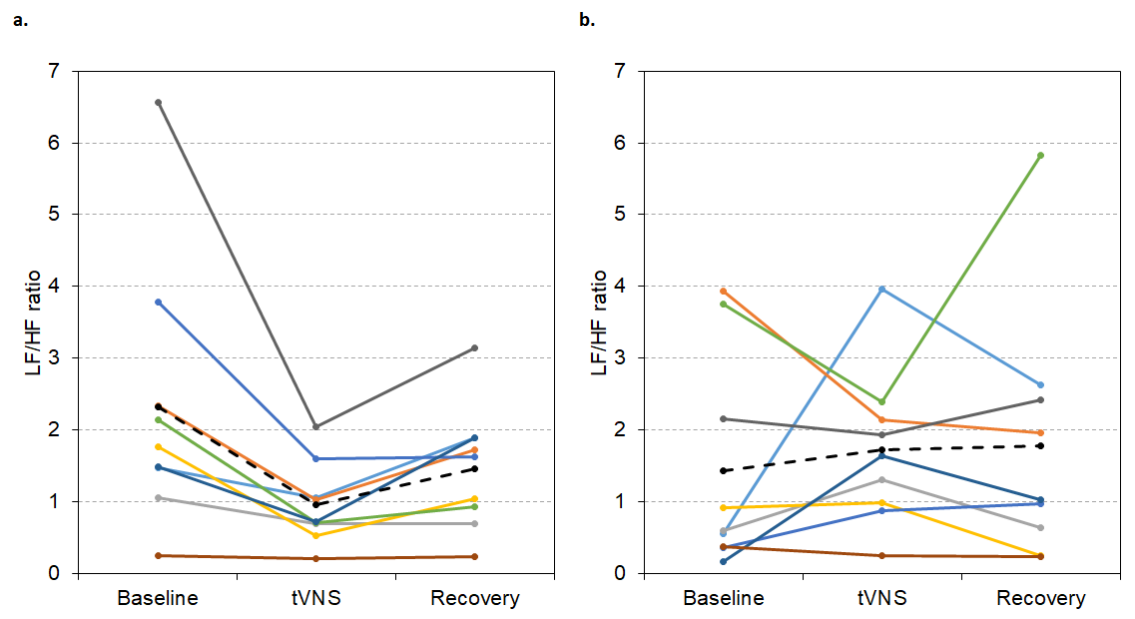
LF/HF ratio values during visit 1 (**A**) and visit 2 (**B**) for each responder during baseline, tVNS and recovery. Dashed black line indicates the group mean.

For the group of responders in visit 1 (n = 9), LF/HF ratio was significantly lower during tVNS (0.95 ± 0.18) compared to baseline (2.32 ± 0.59, p < 0.001) and recovery (1.46 ± 0.27, p = 0.029, Friedman’s test: p = 0.001, see Figure 7a). But, in visit 2, LF/HF ratio did not significantly differ between baseline, tVNS and recovery for the whole group of responders (p > 0.05, see Figure 7b), possibly due to slight decreases in baseline LF/HF ratio.

**Figure 8:**
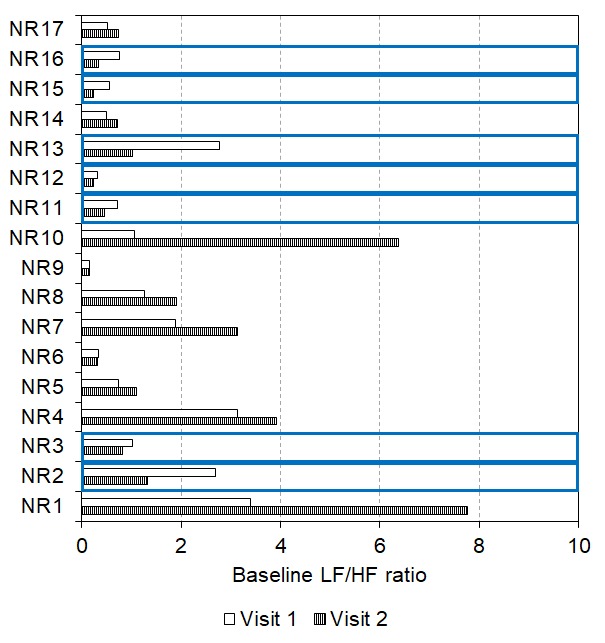
Baseline LF/HF ratio reduced after 2 weeks of daily tVNS in seven non-responders with ten showing an increase in baseline LF/HF ratio following the daily tVNS (indicated by the blue boxes).

Some non-responders (n = 7) even encountered a lowering of their baseline LF/HF ratio after two weeks of daily tVNS (visit 1 mean: 1.26, SEM: 0.39; visit 2 mean: 0.63, SEM: 0.16, p = 0.018, see Figure 8). Seven of these participants then became responders to tVNS in visit 2. In contrast, some non-responders (n = 10) showed increases in baseline LF/HF ratio after two weeks of daily tVNS (visit 1 mean: 1.30, SEM: 0.35; visit 2 mean: 2.61, SEM: 0.80, p = 0.009) all of whom remained non-responders at visit 2.

**Figure 9:**
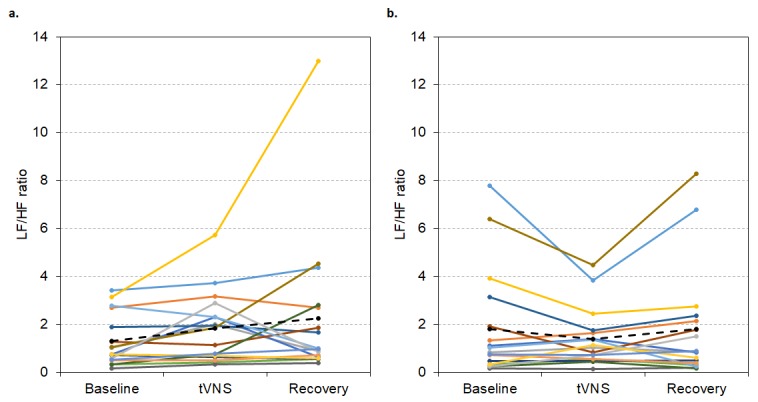
LF/HF ratio values during visit 1 (**A**) and visit 2 (**B**) for each non-responder during baseline, tVNS and recovery. Dashed black line indicates the group mean.

For the group of visit 1 non-responders (n = 17), LF/HF ratio did not significantly differ between baseline, tVNS or recovery in visits 1 or 2 (p > 0.05, see Figure 9), probably due to low initial values.

### Daily tVNS also improved health-related QoL and mood

Aspects of health-related QoL and mood improved following two weeks of daily tVNS (see Supplementary Table 9). For health-related QoL, scores for role limitations due to physical health significantly decreased between visits 1 and 2 (p = 0.026). Additionally, there was a trend for energy scores to increase following the two weeks of daily tVNS (p = 0.058). For mood, self-reported tension (p = 0.015), depression (p = 0.035), vigour (p = 0.030) and mood disturbance (p = 0.006) all significantly improved between the two visits. There was also a trend for self-reported confusion to be lower at visit 2 than at visit 1 (p = 0.059).

**Figure 10:**
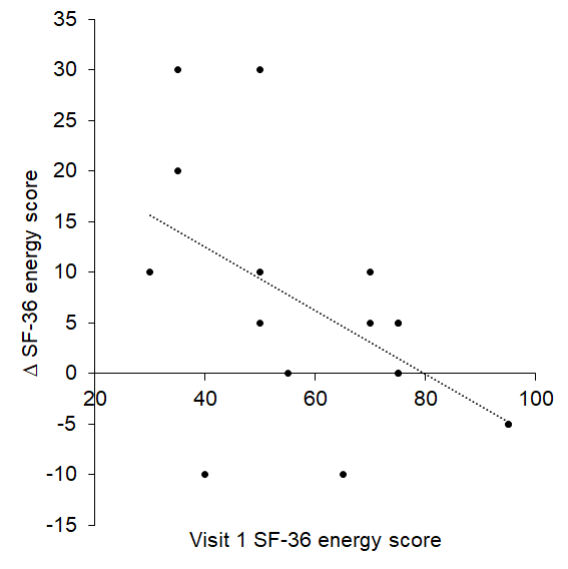
Visit 1 SF-36 energy score significantly predicted change at visit 2.

Linear regressions revealed that SF-36 energy scores during visit 1 significantly predicted change during visit 2 (R^2^ = 0.280, p = 0.029): those with low energy scores at visit 1 reported greater increases at visit 2 (see Figure 10).

**Figure 11:**
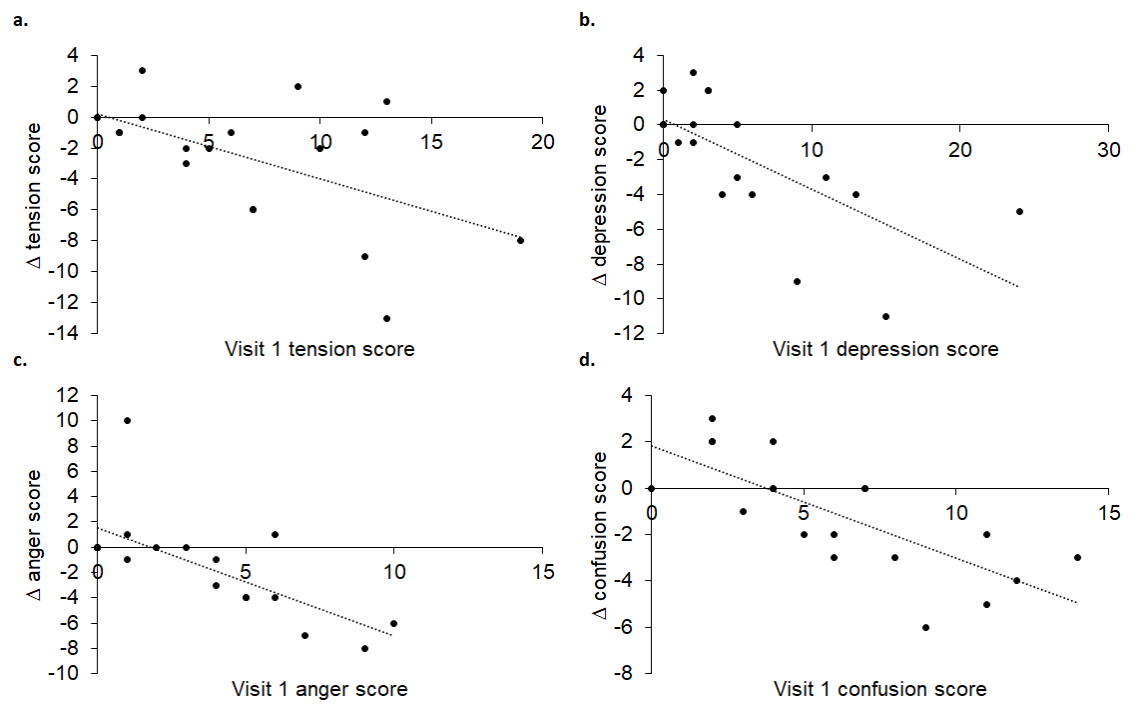
Visit 1 tension (**A**), depression (**B**), anger (**C**) and confusion (**D**) scores significantly predicted change at visit 2.

Furthermore, visit 1 POMS scores for tension (R^2^ = 0.307, p = 0.014), depression (R^2^ = 0.475, p = 0.001), anger (R^2^ = 0.490, p = 0.001) and confusion (R^2^ = 0.560, p < 0.001) significantly predicted scores at visit 2: participants with high tension, depression, anger and confusion scores at visit 1 reported greater improvements at visit 2 than those with low scores (see Figure 11).

**Figure 12:**
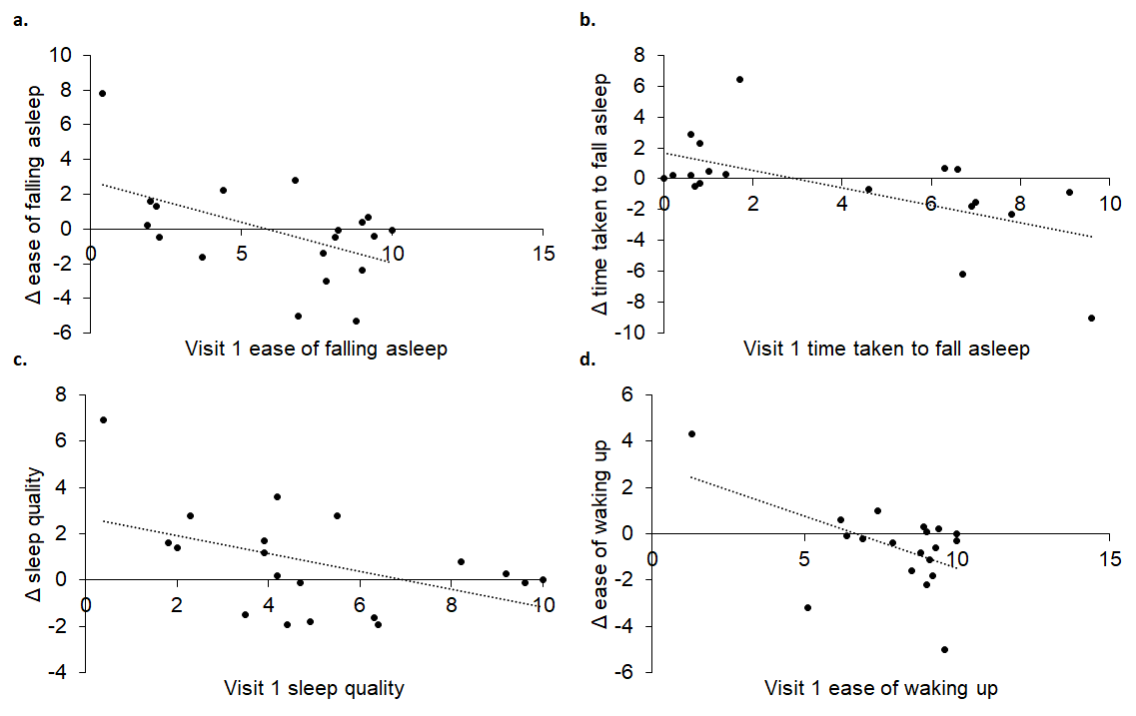
Visit 1 ease of falling asleep (**A**), time taken to fall sleep (**B**), sleep quality (**C**) and ease of waking up (**D**) significantly predicted change at visit 2.

Ease of falling asleep (R^2^ = 0.265, p = 0.024), how quickly it took participants to fall asleep (R^2^ = 0.380, p = 0.005), quality of sleep (R^2^ = 0.223, p = 0.041) and ease of waking up (R^2^ = 0.264, p = 0.024) in visit 1, significantly predicted change between the two visits. As depicted in Figure 12, those who experienced greater improvements in ease of falling asleep, time taken to fall asleep, quality of sleep and ease of waking up at visit 2 had lower values at visit 1.

## DISCUSSION

Transcutaneous vagal nerve stimulation (tVNS) acutely administered to the tragus in healthy volunteers aged ≥ 55 years was associated with improvements in spontaneous cardiac baroreflex sensitivity and HRV. Significantly, tVNS administered for 15 minutes every day for two weeks improved autonomic function and may improve some aspects of health-related QoL, mood and sleep. Intriguingly, findings consistently illustrated that individuals who have greater baseline sympathetic prevalence showed more pronounced shifts towards parasympathetic prevalence with tVNS, with daily tVNS for 2 weeks improving autonomic balance in some individuals. This means that tVNS administered daily for 2 weeks may help attenuate some of the autonomic and psychological changes that occur in ageing, expanding the period of healthy ageing.

### tVNS effects on autonomic function

Positive effects of tVNS on autonomic function have been reported in non-patient groups. For instance, tVNS administered to the tragus significantly reduced sympathetic nerve activity in healthy participants [11] and boosted measures of parasympathetic activity, for example, spontaneous cardiac BRS [12], RMSSD [17], respiratory sinus arrhythmia (coupling between respiration and RR interval [18],) and high frequency power spectrum [19]. Despite this evidence, there is little work examining the autonomic implications of administering tVNS in healthy older individuals who are undergoing age-associated shifts towards sympathetic prevalence. This study therefore examined the autonomic implications of tVNS in individuals aged ≥ 55 years. We anticipated that a single session of tVNS would temporarily improve measures of autonomic function. We also hypothesized that daily administration of tVNS for two weeks would further enhance autonomic tone.

Consistent with [12], a single session of tVNS was associated with greater increases in BRS compared to sham. Furthermore, tVNS promoted shifts towards parasympathetic prevalence in a larger cohort of individuals aged ≥ 55 years with daily tVNS improving resting vagal tone in some participants. BRS is a predictor of all-cause mortality [20]: depressed BRS (< 3 ms/mmHg) was associated with reduced survival rates from cardiovascular death and all-cause mortality. This means that daily tVNS may be an effective tool for strengthening the relationship between changes in RR interval and SBP in individuals who have attenuated BRS, potentially reducing mortality and increasing life expectancy.

Baseline LF/HF ratio (considered a measure of sympathovagal, or autonomic, balance) was a significant predictor of response to tVNS in all three studies: higher LF/HF ratio (greater sympathetic prevalence) was associated with greater decreases in LF/HF ratio (shifts towards parasympathetic prevalence). These patterns emerged in previous work [11], providing further support to potentially using LF/HF ratio as a tool for screening individuals who are likely to encounter greater autonomic benefits from tVNS. Such predictions could enable selection of optimal individuals for tVNS and may be particularly important considering the number of conditions that are characterized with enhanced sympathetic prevalence/autonomic imbalance, e.g. cardiovascular, pain, inflammatory and mental health conditions. Indeed, this would aid with developing a valid inclusion criteria for tVNS studies. Such criteria will need further development - in this study, baseline LF/HF ratios around 1.5 were associated with minimal changes (Δ = 0) whereas those with baseline values > 1.5 were associated with decreases following tVNS. Interestingly, those who had low baseline LF/HF ratios (< 1.5) showed increases following tVNS, presumably due to already low sympathetic and high parasympathetic prevalence. It may therefore be possible to determine baseline LF/HF ratio thresholds which differentiate between response types. However, such thresholds may need to be ascertained with regard to recording and analysis conditions, since HRV can be determined using different algorithms with potentially variable outcomes regarding exact values.

It is particularly interesting that the LF/HF ratio linear regressions reached statistical significance in study 3 where the regression was stronger following two weeks of daily tVNS. Indeed, given the interaction effect between visit and condition for ∆ RR and a stronger linear regression for visit 2, perhaps daily tVNS for two weeks induced some kind of training effect. Furthermore, as some participants showed reductions in baseline LF/HF ratio between the two visits, it seems that daily use of tVNS confers autonomic benefits for some individuals. Indeed, benefits of tVNS delivered over long-time periods have been illustrated elsewhere, although not with regards to autonomic function. For instance, electrical stimulation of ABVN termination sites for 15 minutes per day for two weeks in patients with coronary artery disease decreased the need for vasodilator medication and improved exercise tolerance [21,22]. In addition, in patients with paroxysmal atrial fibrillation (PAF) who received tragus stimulation (active) or ear lobe stimulation (sham) for 1 hour/day for 6 months, there was a 75-85% decrease in AF burden in the active compared to the sham stimulation [23]. Therefore, study 3 provides novel and timely data showcasing that daily tVNS can have profound autonomic benefits in individuals aged ≥ 55 years. Of course, future studies should explore the reproducibility of using baseline LF/HF ratio to determine response type to tVNS. Additionally, it would be of interest to explore the autonomic effects of more long-term use of tVNS and optimum stimulation dosage.

### tVNS effects on quality of life, mood and sleep

As well as conferring autonomic benefits, tVNS also appears to have positive effects on psychological health in patient groups. For instance, tVNS applied for two weeks (once or twice for 15 minutes per day [24],) or for one month (twice for 30 minutes per day [25],) in patients with depression significantly reduced depression scores. Furthermore, tVNS administered for four weeks significantly improved QoL and depression scores in patients with persistent postural-perceptual dizziness [26].

Consistent with the above studies on psychological health, we found that daily tVNS for two weeks significantly improved measures of QoL, in particular, role limitations due to physical health. Furthermore, dimensions of mood including depression, tension, vigour and mood disturbance were all improved following two weeks of daily tVNS. These findings therefore suggest that daily tVNS may be an effective means of improving aspects of everyday life in this age group. However, it should be acknowledged that since this was a single-arm study, it is possible that these self-report measures could have been influenced by a placebo effect. Therefore, to further explore the effect of daily tVNS on subjective measures (such as QoL and mood), future studies should embrace designs and protocols which allow for an assessment of the contribution of placebo effects to improvements in such measures, perhaps via a significantly enlarged sample size. This could also be combined with other measures which assess performance on cognitive tasks. This would be particularly interesting, given that cognitive function declines in older age and studies have shown that tVNS can boost divergent thinking [27], associative memory in older individuals [28] and the recognition of emotions in faces when presented with whole faces [29] and just the eye region [30].

Sleep has also been shown to deteriorate with age [31] and could therefore potentially benefit from daily tVNS. Indeed, on returning for their second visit, some participants commented on improvements in aspects of their sleep. Indeed, participants who found that falling asleep took a long time and was difficult, had low sleep quality and had difficulties waking up in the morning, showed great improvements following two weeks of daily tVNS. This is an important finding and warrants further investigation employing a control group along with validated sleep questionnaires, such as the Pittsburgh Sleep Quality Index and polysomnography.

### Age-related conditions which could benefit from tVNS

Normal ageing is associated with increases in sympathetic prevalence and/or decreases in vagal tone and overall variability [1,2]. These ageing effects interact with gender, whereby the greater sympathetic prevalence in young males compared to young females disappears in older age [2]. In addition to normal ageing, shifts towards sympathetic prevalence may contribute to age-related conditions, for example, hypertension, heart failure and atrial fibrillation. Evidence suggests that tVNS could play a role in ameliorating these conditions.

Electrical stimulation of ABVN termination sites for only 15 minutes per day for 14 days in patients with coronary artery disease decreased the need for vasodilator medication and improved exercise tolerance [21,22]. Furthermore, tVNS reduced atrial fibrillation in patients with paroxysmal atrial fibrillation (PAF) and reduced plasma levels of the inflammatory cytokine TNFα [32], which is associated with chronic increases in inflammation with ageing [33]. Similar positive effects were observed when low-level tragus stimulation (LL-TS) was administered to canines [34]. Indeed, LL-TS successfully reversed an increase in neural activity (in the anterior right ganglionated plexi) and a decrease in the window of vulnerability and effective refractory period that accompanied induced atrial fibrillation [34]. Therefore, tVNS administered to ABVN sites appears to temporarily improve symptoms associated with age-related conditions, such as PAF and coronary artery disease.

In addition to age-associated cardiovascular pathophysiology, tVNS may confer benefits in other age-related conditions. Also, tVNS reduced pain ratings in chronic pelvic pain due to endometriosis in females [35] and during sustained application of painful heat [36]. These findings are noteworthy given that ageing is associated with increases in pain [37]. tVNS may even be used as a non-invasive functional technique for the early diagnosis of Alzheimer’s disease and other neurodegenerative conditions [38] and aid with the progression of obesity and type 2 diabetes [39–42].

Considering the ease of application and affordability of tVNS there is significant potential in prolonging the period of healthy ageing and attenuating symptoms associated with age-related conditions. Of course, considerable work exploring the optimum tVNS stimulation parameters (current, pulse width, pulse frequency), tVNS session duration (e.g. 15 minutes) and chronic paradigm (e.g. once daily for two weeks) for specific conditions/patient groups may be required. Future studies should also carefully consider their study design, especially with respect to control groups. Indeed, it should be acknowledged that the single-arm design of studies 2 and 3 is a limitation. However, this design was adopted for two main reasons. Firstly [11], and study 1 revealed tVNS was associated with greater autonomic balance compared to sham. Secondly, there are challenges with establishing a valid control for chronically administered tVNS. For instance, if the sham arm comprised of altering the internal workings of the machine so that it administered no electrical impulses but the machine still ‘looked’ active, participants might have realized they were receiving no stimulation (given that tVNS is perceptible). In which case, the sham would not have been able to explore the contribution of a placebo effect to changes in measures of autonomic function, health-related QoL, mood and sleep. An alternative sham could have entailed administering the stimulation to the earlobe. However, the assertion that the earlobe is not vagally innervated is based on one study [43] and there has recently been controversy about the anatomical location of the auricular vagus [44–46]. Due to these challenges, we decided to implement a single-arm design for studies 2 and 3 where each participant acted as their own control. Future work could implement a wash-out period, which would also aid with ascertaining the time required for the effect of daily tVNS to diminish. This future work should also explore the extent to which the changes in the self-report measures were due to tVNS, a placebo effect or a participant bias.

## CONCLUSION

For the first time, we have shown that age-related autonomic, QoL, mood and sleep changes may be improved with tVNS administered every day for two weeks. Importantly, the findings point to the influence of initial values in determining magnitude and direction of change following tVNS: high initial sympathetic prevalence, tension, depression, anger and confusion and low energy and sleep quality were associated with greater improvements. With further work, it may therefore be possible to identify which individuals will most benefit from daily tVNS in terms of their autonomic function and overall well-being.

## METHODS

### Ethical approval

University of Leeds ethical approval was secured (Ethics Reference: BIOSCI 13-025) and the study conformed to the standards outlined in the Declaration of Helsinki. Informed written consent was obtained voluntarily by all research participants and their data were anonymised and stored securely according to the UK Data Protection Act (1998). Participants were informed that they could withdraw from the experiment at any time.

### Participants

Participants were excluded if they had a history of cardiovascular disease, epilepsy or episodes of frequent fainting (syncope). Participants were asked to abstain from caffeine, alcohol, nicotine and strenuous exercise for a minimum of 12 hours prior to their visit. No female participants who participated were taking hormone replacement therapy (HRT).

For study 1, 14 healthy volunteers aged 55 years or over were recruited (n = 9 males, mean age: 69.11 ± 1.52 years). An additional 37 healthy participants aged 55 years or over were recruited for study 2, resulting in a total sample size of 51 participants (n = 24 males, mean age: 65.20 ± 0.79 years). The daily tVNS study was conducted on 29 participants (study 3, n = 11 males, mean age range: 64.14 ± 0.89 years).

### Procedure

All study visits were carried out in a quiet, temperature controlled (21 ± 2°C) human physiology study room at the University of Leeds between the hours of 09.00 and 12.00. Participants reclined semi-supine on a couch for the duration of each experiment.

### Transcutaneous vagus nerve stimulation (tVNS)

tVNS was performed using a TENS machine (V-TENS Plus, Body Clock Health Care Ltd, United Kingdom in studies 1 and 2 and EMS7500 Roscoe Medical in study 3) with customised auricular electrode clips attached on the inner and outer surface of the tragus of the ear (Auricular Clips, Body Clock Health Care Ltd, UK). Participants wore the electrode clips throughout all three recordings (baseline, stimulation, recovery). tVNS was applied continuously for 15 minutes with a pulse width of 200 µs and pulse frequency of 30 Hz. Amplitude was adjusted to the level of sensory threshold (usually 2-4 mA) until the participants reported a ‘pin-prick’ or ‘tingling’ sensation. The stimulus was then turned down until the stimulus was borderline perceptible and comfortable.

### Study 1 procedure

**Figure 13:**
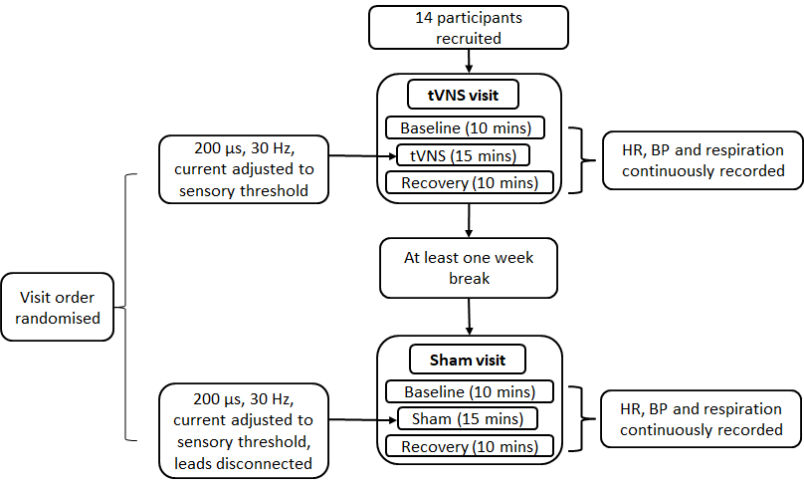
**Procedure for study 1.**

Study 1 examined the extent to which tVNS and sham stimulation impacted measures of autonomic function. Participants attended a ‘tVNS visit’ and a ‘sham visit’. Sham stimulation entailed positioning the surface electrodes as per tVNS and informing participants that a different set of stimulation parameters would be tested involving a reduction in the current below the participant’s level of sensory perception. The electrode leads were then disconnected from the TENS machine without the participant’s knowledge. The order of the visits was randomized between participants. At the beginning of each visit, they completed a basic health questionnaire, physical activity level was assessed using the Godin Leisure Time Exercise Questionnaire [47] and height and weight obtained. Physiological equipment that continuously recorded HR, blood pressure (BP) and respiration were then attached and participants rested for 10 minutes before recordings commenced. Three sets of recordings were obtained: 10 minute baseline, 15 minute stimulation and 10 minute recovery. The order of the recordings was identical for all participants. Figure 13 summarises the procedure employed in study 1.

### Study 2 procedure

**Figure 14:**
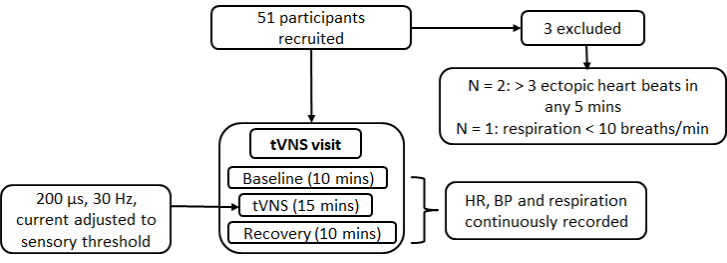
**Procedure for study 2.**

Study 2 explored the effects of acute tVNS (i.e. a single 15-minute session) on autonomic function in a larger group of individuals aged ≥ 55 years. The procedure was identical to that employed in study 1, with the exception that participants were not required to attend for a sham visit (rationale based on study 1 results). Figure 14 summarises the study 2 procedure.

### Study 3 procedure

**Figure 15:**
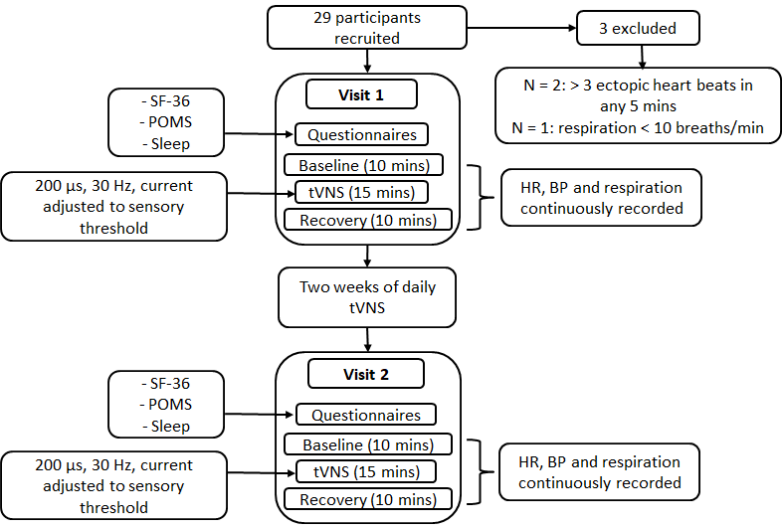
**Procedure for study 3.**

Study 3 examined the effects of daily tVNS on autonomic function, QoL, mood and sleep in 29 healthy volunteers aged ≥ 55 years (Figure 15). The procedure was similar to that employed in study 1, with the exception that participants attended on a second occasion, exactly two weeks following their first visit for a second tVNS visit. In addition, participants completed the SF-36, POMS questionnaire (Profile of Mood States) and a sleep questionnaire at the beginning of each visit. At the end of their first visit, participants were provided with a tVNS machine to take home. They were trained in how to use the device and instructed to do a 15-minute session once daily for two weeks. A log sheet was provided so that participants could record device usage and any comments/observations following each session.

### Measurements

In all three studies, HR, non-invasive BP and respiration were continuously recorded during the three recordings (baseline, tVNS, recovery). Non-invasive BP was recorded for the primary purpose of deriving BRS. In study 3, the SF-36 (measuring health-related QoL), Profile of Mood States (POMS) questionnaire (measuring mood) and sleep questionnaire (measuring sleep quality, ease of falling asleep and waking up, and sleep interruption) were completed by participants. Frequency-domain, time-domain and non-linear HRV and cardiac BRS were derived for the final five minutes of each recording and health-related QoL, mood and sleep scores were also calculated for study 3. For further information on the measurements obtained, please see the Methods Supplementary Section.

### Data analysis

The final five minutes of each recording were analysed offline in LabChart 8 (AD Instruments). Frequency-domain, time-domain and non-linear HRV were derived along with BRS. Change (Δ) between baseline and stimulation was calculated in all three studies.

### HRV variables

#### Frequency-domain HRV

Frequency-domain HRV parameters included: the low frequency (LF) component, detected at 0.04-0.15 Hz; the high frequency (HF) component, detected at 0.15-0.40 Hz; total power (0.04-0.40 Hz) and normalised values for LF and HF power (nuLF and nuHF). The HF component has been associated with parasympathetic modulation of heart rate [48], whereas LF power reflects both parasympathetic and sympathetic modulation of heart rate [49]. The ratio of LF to HF power (LF/HF) was also calculated along with normalised LF/HF (nuLF/HF) where baseline values were set to 1. A decrease in LF/HF ratio suggests a shift in cardiac autonomic input towards either reduced sympathetic activity and/or increased vagal parasympathetic activity.

#### Time-domain HRV

Five time domain HRV variables were analysed: mean RR interval, Δ RR (difference between the longest and shortest RR intervals), SDRR (standard deviation of all RR intervals), RMSSD (the square root of the squared of differences between adjacent RR intervals) and pRR50 (percentage of number of pairs of adjacent RR intervals differing by more than 50 ms). SDRR reflects global autonomic regulation and is an estimate of all HRV [50]. RMSSD and pRR50, being mainly related to beat-to-beat variations, reflect parasympathetic output [50].

#### Non-linear HRV

The Poincaré plot is a two-dimensional graphic representation of the correlation between consecutive RR intervals, in which each interval is plotted against the following interval. For quantitative analysis, an ellipse is fitted to the shape formed by the plot with the center determined by the average RR intervals. Two parameters were derived: SD1 and SD2. SD1 measures the standard deviation of the distances of the points to the diagonal y=x and SD2 measures the standard deviation of the distances of points to the line y=-x + RRm, where RRm is the average of RR intervals. SD1 is an index of instantaneous recording of the variability of beat-to-beat and represents parasympathetic activity, while SD2 represents the variability of long and short term variability. SD1 and SD2 were normalised relative to heart rate by computing: (SD1 or SD2/RR interval)*1000. SD2/SD1 was also derived and represented the ratio between short and long term variations in RR intervals. The area of the ellipse, S, representing total variability in RR intervals was ascertained by calculating π*SD1*SD2.

### Cardiac baroreflex sensitivity (BRS)

Cardiac BRS was assessed using the sequence method, in which ‘up’ and ‘down’ sequences were identified [51]. ‘Up’ sequences consisted of three or more consecutive cardiac cycles for which there was a sequential rise in both SBP (≥ 1 mmHg) and RR interval (≥ 2 ms). ‘Down’ sequences consisted of three or more cardiac cycles for which there was a sequential fall in SBP and RR interval.

In Excel 2013, the RR interval was plotted against SBP for each sequence (r ≥ 0.85 acceptance level) and the average slope values for the ‘up’ and ‘down’ sequences were combined to get an average cardiac baroreflex slope. Values of cardiac BRS were accepted when the number of sequences was ≥3 for both up and down sequences.

### Statistical Analysis

Statistical analysis was performed using SPSS (version 25). Normality of distribution was tested using Shapiro-Wilk. Two-tailed statistical tests were used in all instances with an alpha level of 0.05. All data are presented as group mean ± standard error of the mean (SEM) unless otherwise stated.

The supplementary methods and materials section provides further information about the statistical tests performed. In brief, paired sample t-tests (or Wilcoxon signed-rank tests) explored differences between tVNS and sham (study 1) and following 14 days of tVNS (study 3). Repeated measure ANOVAs examined differences between recordings (study 2) and visits (study 3, two-way repeated measure ANOVAs). Linear regressions explored the extent to which baseline autonomic function significantly predicted response to a single session and daily use of tVNS.

## SUPPLEMENTARY MATERIAL

Supplementary Methods

Supplementary Tables
