# Supplementary material for: Effects of transcutaneous vagus nerve stimulation in individuals aged 55 years or above: potential benefits of daily stimulation
Source: Aging (Albany NY). 2019 Jul 30;11(14):4836–57. doi: 10.18632/aging.102074 (PMC6682519; doi:10.18632/aging.102074)
Supplement: Supplementary Methods [file aging-11-102074-s001.pdf]

SUPPLEMENTARY MATERIAL

Supplementary Methods

Measurements

Heart rate was derived from electrocardiogram (ECG) recorded by 3 Ag-AgCl surface electrodes on the chest sampled at 2 kHz and continuous blood pressure was recorded via finger pulse plethysmography (Finometer; Finapres Medical System, Netherlands) sampled at 400 Hz in LabChart 8 (AD Instruments). Continuous blood pressure recordings were obtained in order to derive cardiac baroreflex sensitivity (BRS). Respiration was recorded via a strain-gauge transducer (Pneumotrace, UFI, CA, USA) placed around the chest and sampled at 0.4 kHz (in LabChart 8). Participants were allowed to breathe spontaneously but their respiration rate monitored to ensure there were no major deviations ($> \pm 2$ breaths per minute from baseline value) and a minimum respiration rate of ≥ 10 breaths per minute was met. One participant in studies 2 and 3 had a respiration rate < 10 breaths per minute and was subsequently excluded from all further analyses.

In study 3, two questionnaires were completed by participants at the beginning of each visit: SF-36 and Profile of Mood States (POMS). The SF-36 provides an indication of health-related QoL from which scores on different aspects of QoL can be derived e.g. physical functioning, role limitations due to physical and emotional health, energy, emotional well-being, social functioning, pain, and general health scores. The POMS questionnaire was used to gain an indication of how specific aspects of mood may change as a result of the fortnight of daily tVNS e.g. depression, tension, anger, fatigue, confusion, vigour and mood disturbance).

Statistical Analysis

Study 1

To assess the extent to which autonomic function (HRV and BRS) was significantly influenced by tVNS and sham stimulation, paired sample t-tests (or Wilcoxon signed-rank tests) were performed on change (Δ) between baseline and stimulation. Linear regressions examined whether baseline measures of autonomic function significantly predicted response (change (Δ) between baseline and stimulation).

Study 2

To explore the extent to which tVNS significantly influenced measures of autonomic function in a larger sample of individuals aged ≥ 55 years, one-way repeated measure ANOVAS with Bonferroni pairwise comparisons were conducted. For non-normally distributed data, Friedman tests with Wilcoxon signed-rank post-hoc tests were performed. Linear regressions explored the extent to which baseline measures of autonomic function significantly predicted response (Δ) to tVNS. To explore differences in baseline measures of autonomic function, independent sample t-tests (or Mann-Whitney U tests) were performed.

Study 3

Paired sample t-tests (or Wilcoxon signed-rank tests) examined the extent to which baseline autonomic tone, health-related QoL, mood and sleep changed following the fortnight of daily home-use tVNS. Two-way repeated measure ANOVAs explored differences in measures of autonomic function between the two visits and between the three conditions. Linear regressions assessed whether baseline autonomic tone, health-related QoL, mood and sleep in visit 1 predicted change to visit 2 baseline to tVNS. Independent sample t-tests (or Mann-Whitney U tests) explored differences between responders and non-responders.
