# Supplementary material for: Effects of transcutaneous vagus nerve stimulation in individuals aged 55 years or above: potential benefits of daily stimulation
Source: Aging (Albany NY). 2019 Jul 30;11(14):4836–57. doi: 10.18632/aging.102074 (PMC6682519; doi:10.18632/aging.102074)
Supplement: Supplementary Tables [file aging-11-102074-s002.pdf]

Supplementary Tables

Study 1

Supplementary Table 1. Summary of characteristics of the final sample of study 1.

Final sample size (n)	14
Gender (frequency of males)	9
Age (yrs.)	69.14 (1.82)
BMI (kg/m^2)	25.83 (1.05)
Baseline SBP (mmHg)	121.07 (3.87)
Baseline DBP (mmHg)	74.29 (1.80)
Baseline MAP (mmHg)	89.88 (2.42)

Study 2

Supplementary Table 2. Summary of characteristics of the final sample of study 2.

Final sample size (n)	48
Gender (frequency of males)	22
Age (yrs.)	65.28 (0.85)
BMI (kg/m^2)	27.13 (0.79)
Baseline SBP (mmHg)	123.63 (2.44)
Baseline DBP (mmHg)	79.09 (1.42)
Baseline MAP (mmHg)	93.94 (1.69)

Supplementary Table 3. Statistically significant differences between the three recordings (baseline, tVNS, recovery) transpired. Reported p-values are from the one-way ANOVAs/Friedman tests. * = significantly different to baseline (post-hoc tests). Data presented as the mean ± 1 SEM.

	Baseline	tVNS	Recovery	p-value
Total power (ms^2)	1161 (145)	* 1726 (255)	* 2011 (259)	0.007
LF power (ms^2)	284 (51)	* 391 (75)	* 460 (61)	0.009
HF power (ms^2)	351 (68)	388 (69)	* 448 (85)	0.001
nuLF	51 (3)	52 (3)	55 (3)	n.s
nuHF	50 (3)	49 (2)	46 (3)	n.s
LF/HF ratio	1.58 (0.21)	1.40 (0.16)	1.96 (0.32)	n.s
Mean RR interval (ms)	963 (20)	* 1001 (21)	* 1009 (21)	< 0.001
Δ RR (ms)	225 (16)	* 249 (15)	244 (14)	< 0.001
SDRR (ms)	35 (2)	* 42 (3)	* 42 (3)	< 0.001
RMSSD (ms)	28 (3)	* 31 (3)	31 (3)	< 0.001
pRR50 (%)	9 (2)	* 10 (2)	10 (2)	0.003
SD1 (ms)	20 (2)	* 22 (2)	22 (2)	< 0.001
SD2 (ms)	44 (2)	* 54 (4)	* 55 (3)	< 0.001
nSD1	20 (2)	21 (2)	21 (2)	n.s
nSD2	46 (2)	* 53 (3)	* 55 (3)	0.006
SD2/SD1	2.83 (0.21)	2.82 (0.18)	2.93 (0.17)	n.s
S (ms^2)	3258 (507)	* 4586 (794)	* 4429 (660)	< 0.001
BRS (ms/mmHg)	7.22 (0.60)	* 8.83 (0.67)	8.29 (0.67)	0.006

Supplementary Table 4. Summary of statistically significant differences in baseline measures of autonomic function between responders and non-responders. Data presented as the mean ± 1 SEM.

	Responders (n = 16)	Non-responders (n = 32)	p-value
HF power (ms^2)	179.98 (59)	436.47 (95)	0.013
nuLF	65.88 (4)	43.94 (4)	0.001
nuHF	35.43 (4)	56.93 (4)	0.001
LF/HF ratio	2.70 (0.42)	1.09 (0.17)	< 0.001
RMSSD (ms)	22.11 (5)	31.39 (3)	0.029
SD1 (ms)	15.66 (3)	22.17 (2)	0.029
nSD1	16.67(3)	22.01 (2)	0.029
SD2/SD1	3.53 (0.46)	2.48 (0.20)	0.047
S (ms^2)	2548.37 (927)	3612.35 (604)	0.034
BRS (ms/mmHg)	5.15 (0.93)	8.32 (0.71)	0.011

Supplementary Table 5. Demographic characteristics of responders and non-responders: there were no statistically significant differences between responders and non-responders.

	Responders (n = 16)	Non-responders (n = 32)	p-value
Gender (male)	6	16	n.s
Age (yrs.)	66.63 (1.87)	64.56 (0.84)	n.s
BMI (kg/m^2)	27.51 (0.98)	26.95 (1.08)	n.s

Study 3

Supplementary Table 6. Measures of vagal tone were significantly higher during visit 2 compared to visit 1. Reported p-values are from the significant main effects of visit. Data presented as the mean ± 1 SEM.

	Visit 1	Visit 2	p-value
Total power (ms^2)	1618 (264)	2202 (389)	0.046
HF power (ms^2)	443 (107)	676 (146)	0.051
SDRR (ms)	40 (4)	45 (4)	0.024
RMSSD (ms)	30 (4)	36 (5)	0.016
pRR50 (%)	11 (3)	15 (4)	0.010
SD1 (ms)	21 (3)	26 (3)	0.016
nSD1	20 (2)	25 (3)	0.022
SD2 (ms)	52 (4)	57 (5)	0.035
S (ms^2)	4189 (830)	5828 (1099)	0.012

Supplementary Table 7. Measures of overall variability significantly differed between the three recordings (baseline, tVNS, recovery). Reported p-values are from the significant main effects of recording. * = significantly different to baseline. Data presented as the mean ± 1 SEM.

	Baseline	tVNS	Recovery	p-value
Total power (ms^2)	1345 (205)	* 2216 (395)	* 2169 (400)	0.010
LF power (ms^2)	325 (54)	* 529 (109)	544 (116)	0.039
SDRR (ms)	36 (3)	* 47 (5)	* 44 (4)	< 0.001
SD2 (ms)	45 (3)	* 61 (6)	* 57 (5)	< 0.001
S (ms^2)	3814 (731)	* 5876 (1244)	5336 (1035)	0.026
Mean RR interval (ms)	970 (32)	* 1011 (33)	* 1010 (33)	< 0.001
nSD2	46 (3)	* 59 (5)	* 56 (4)	< 0.001

Supplementary Table 8. Summary of demographic characteristics and visit 1 baseline autonomic measures for responders and non-responders. Data are presented as the mean ± 1 SEM.

		Responders (n = 9)	Non-responders (n = 17)	p-value
Demographic characteristics	Gender (male)	3	6	n.s
	Age (yrs.)	63.78 (1.52)	64.29 (1.29)	n.s
	BMI (kg/m^2)	28.12 (1.70)	28.05 (1.73)	n.s
Visit 1 baseline autonomic measures	HF power (ms^2)	197.23 (99)	528.75 (166)	0.034
	SDRR (ms)	26.45 (4)	39.21 (4)	0.038
	SD2 (ms)	33.92 (4)	49.65 (18)	0.028
	nSD1	14.57 (4)	22.83 (3)	0.039
	nSD2	36.22 (4)	51.08 (4)	0.036
	S (ms^2)	1762.81 (611)	4128.50 (1004)	0.039
	BRS (ms/mmHg)	4.35 (1.19)	8.24 (1.11)	0.036

Supplementary Table 9. Summary of changes in health-related QoL and mood between visits 1 and 2. Data presented as the mean ± 1 SEM.

		Visit 1	Visit 2	p-value
SF-36	Role limitations due to physical health score	84 (7)	69 (11)	0.026
	Energy score	61 (5)	67 (4)	0.058
POMS	Tension score	7 (1)	4 (1)	0.015
	Depression score	6 (2)	3 (1)	0.035
	Vigour score	19 (1)	21 (1)	0.030
	Mood disturbance score	8 (5)	-2 (5)	0.006
	Confusion score	6 (1)	5 (1)	0.059
